# Empowering breast cancer clients through AI chatbots: transforming knowledge and attitudes for enhanced nursing care

**DOI:** 10.1186/s12912-025-03585-w

**Published:** 2025-07-29

**Authors:** Mostafa Shaban, Yasmine M. Osman, Nermen Abdelfatah Mohamed, Marwa Mamdouh Shaban

**Affiliations:** 1 Lecturer of Geriatric Nursing, Faculty of Nursing, Cairo University, Cairo, Egypt; 2 Department of Obstetrics and Gynecology Nursing, Faculty of Nursing, Zagazig University, Zagazig, Egypt; 3 Present Address: Assistant professor of Adult Nursing Department, Faculty of Nursing, Kafr Elsheikh University, Kafr Elsheikh, Egypt; 4 Lecturer of Community Health Nursing- Faculty of Nursing- Cairo University, Cairo, Egypt

**Keywords:** Breast cancer, AI chatbot, Empowerment, Patient education, Attitudes, Randomized controlled trial

## Abstract

**Background:**

Breast cancer remains a leading cause of morbidity worldwide, necessitating innovative and accessible interventions that address both clinical and psychosocial needs. AI-powered chatbots are increasingly used in health education due to their 24/7 availability, personalization, and interactivity. However, empirical evidence on their effectiveness in enhancing knowledge, empowerment, and attitudes in oncology settings remains limited.

**Aim:**

This randomized controlled trial (RCT) evaluated the impact of an AI chatbot intervention on knowledge, empowerment, and attitudes toward AI among breast cancer patients.

**Methods:**

A two-arm, pre–post RCT was conducted with 122 women diagnosed with breast cancer at Kafr El-Sheikh University Hospital. Participants were randomly assigned to an intervention group (*n* = 61) receiving structured AI chatbot-based education plus standard care, or a control group (*n* = 61) receiving standard care alone. Data were collected using validated questionnaires assessing breast cancer and AI knowledge, attitudes toward AI, and perceived empowerment. G*Power analysis determined sample adequacy for between-group comparisons.

**Results:**

Post-intervention, the intervention group showed significantly higher knowledge (20.3 ± 2.1 vs. 17.9 ± 3.4, *p* <.001) and more positive attitudes (82.4 ± 7.2 vs. 72.6 ± 8.9, *p* <.001) compared to controls. Logistic regression indicated that knowledge gain and higher education predicted a positive AI attitude. Path analysis revealed both direct and mediated effects of knowledge on attitude via empowerment. Usage data and chatbot session logs supported high engagement.

**Conclusion:**

Integrating AI chatbots into oncology nursing care significantly enhances knowledge, empowerment, and AI acceptance. These findings support chatbot integration in patient-centered digital health strategies, particularly in oncology.

**Clinical trial number:**

Not applicable.

**Trial registration:**

NCT06943911 (retrospectively registered on 24/4/2025).

**Supplementary Information:**

The online version contains supplementary material available at 10.1186/s12912-025-03585-w.

## Introduction

Breast cancer is still one of the most common cancers in the world, imposing enormous physical, emotional, and financial costs on patients and their families [1]. Despite breakthroughs in identification and treatment, many patients continue to experience substantial psychological discomfort, feelings of isolation, and a lack of understanding of their available treatment choices [2, 3]. Globally, healthcare systems try to not only prolong but also enhance patients’ quality of life, emphasising the significance of psychological assistance in addition to medical intervention [4]. Nursing care, which combines professional knowledge and compassionate interaction, is critical in navigating patients through the complexity of breast cancer diagnosis and treatment [5]. Even with the best efforts of dedicated healthcare professionals, the growing number of patients and the need for consistent support highlight a pressing concern: traditional, face-to-face education and counselling sessions may not be adequate to meet the diverse needs of all breast cancer patients [6].

The growth of digital technology in healthcare has ushered in a new era of patient interaction, with information available swiftly and easily across several platforms. These improvements are especially important for cancer patients, who often demand rapid, accurate, and personalised information to comprehend their diagnosis, deal with treatment side effects, and make educated choices [7]. AI-powered chatbots are among these revolutionary digital technologies, which use algorithms and natural language processing to replicate human communication [8, 9]. Over the last decade, chatbots have evolved from basic question-and-answer bots to complex systems capable of providing tailored health information, symptom triage, and psychological support [10]. Despite the rising complexity of chatbots and increased consumer adoption of digital health technologies, research into how AI chatbots might empower breast cancer patients remains restricted [11].

Breast cancer treatment is extremely personal and varied, with medical, emotional, and social components that may be particularly difficult for patients. In this context, the idea of using AI chatbots to deliver personalised guidance and credible instructional resources is quite promising [12]. A well-designed AI chatbot may be taught to recognise user enquiries about symptoms, treatment methods, or lifestyle changes, and provide quick, evidence-based solutions [13]. Furthermore, these chatbots may be connected into current healthcare systems, allowing them to offer individualised messages based on each patient’s medical history and treatment path [14, 15]. From a patient’s viewpoint, chatbot exchanges may take place outside of normal hospital hours, addressing knowledge gaps and emotional distress that may emerge when urgent human aid is unavailable [16]. This combination of technology and human-centered design has the potential to reduce patient anxiety, reinforce treatment adherence, and even promote proactive health behaviours [17].

Despite these benefits, the research demonstrates gaps in our understanding of how AI chatbots affect the knowledge base and attitudes of breast cancer patients. Many contemporary digital health initiatives focus on either patient education or psychological support, without properly investigating how these two areas interact when enabled by artificial intelligence [18]. Furthermore, while some studies show that chatbot interfaces are generally well-liked [19], more research is needed into how such AI-powered platforms can change patient attitudes, more research is needed into how such AI-powered platforms can change patient attitudes towards treatment, foster greater self-efficacy, and cultivate partnerships between patients and healthcare professionals [20]. Trust, privacy, and the authenticity of chatbot discussions remain issues, posing a challenge to the tools’ acceptability and long-term usage. These unanswered problems highlight the urgent need for extensive study that not only investigates the technological viability of AI chatbots, but also assesses their psychological and behavioural implications [21, 22].

Against this context, nursing care is well positioned to pioneer the use of AI chatbots into breast cancer treatment tactics. Nurses often serve as patients’ initial point of contact when they need advice, comfort, or clarity regarding their condition [23]. Incorporating AI chatbots into healthcare processes may reduce the workload on nursing staff, enabling them to concentrate on more complicated or urgent patient requirements while ensuring that simple enquiries and concerns are immediately answered [24]. To ensure the success of such integration, evidence-based frameworks identifying the possible advantages, constraints, and best practices for chatbot usage in oncology settings must be established [25]. This endeavour necessitates an interdisciplinary strategy in which nursing science, artificial intelligence, and psychosocial oncology collaborate to develop patient-centered solutions [26].

Early research in this sector suggests that providing patients with timely and credible educational materials on their diagnosis and treatment plan might reduce anxiety while increasing a feeling of control [27]. However, there has been little research into the processes by which AI chatbots affect patient attitudes and health behaviours. For example, it is unknown if a chatbot’s conversational style can successfully establish rapport or empathy, both of which are critical components in nursing encounters [28, 29].

AI-powered chatbots were selected as the optimal intervention due to their ability to provide interactive, real-time, and personalized support—advantages that static websites and traditional mobile apps often lack. Unlike pre-programmed digital platforms, AI chatbots can simulate human dialogue, adapt responses to user queries, and maintain ongoing engagement through conversational interfaces. These tools offer 24/7 availability, which is particularly valuable for patients facing anxiety, uncertainty, or practical concerns outside clinical hours. Additionally, chatbots can deliver tailored information based on patient profiles, ensuring relevance and accessibility, especially for individuals with varying literacy or health literacy levels. In contrast to conventional web-based education tools, the immediacy and conversational nature of chatbots foster a more dynamic and supportive user experience, aligning with the needs of oncology patients who require both emotional reassurance and accurate medical information.

## Materials and methods

### Research design

This research used a randomized controlled trial (RCT) with a pre–post-intervention design to assess the impact of AI chatbots on the knowledge and attitudes of breast cancer patients. This approach was chosen for its appropriateness in assessing the effects of an intervention in practical environments without requiring randomization. The main objective was to evaluate changes in participants’ comprehension of their condition and their attitudes about AI technology in healthcare after interacting with a customized AI-driven chatbot. The pre-and post-intervention technique facilitated the gathering of baseline data, execution of the intervention, and subsequent assessment of results, therefore assuring a rigorous and systematic approach to evaluating the intervention’s success.

### Setting

The research was carried out at the oncology outpatient clinics of Kafr El-Sheikh University Hospital, a top hospital in the area known for its thorough cancer treatment programs. Offering a broad spectrum of diagnostic and therapeutic treatments, the clinics attract a varied patient population from urban and rural locations. This location was selected as it provides direct access to breast cancer patients actively involved in their treatment path, therefore offering a perfect scenario for researching the integration of creative technology into patient care. Furthermore, the familiarity and confidence consumers have with the institution improved involvement and recruiting.

### Sample and sampling

To ensure that the chosen sample was both representative of the target population of the study and in line with the research aims, the study used a purposive sampling approach to identify individuals who satisfied certain inclusion criteria. This approach was used as it let one deliberately choose people most likely to help and provide insightful analysis of the intervention. The research was carried out at Kafr El-Sheikh University Hospital’s oncology outpatient clinics, which yearly handle about 900 breast cancer patients. Based on qualifying criteria and computed sample size, 122 people were gathered from this overall population.

### Sample size calculation

The sample size was determined using G-Power software (version 3.1.1) to ensure the study was adequately powered to detect meaningful differences in outcomes. The parameters for the calculation were as follows:

- **Effect size (Cohen’s d)**: 0.3, representing a moderate effect size commonly observed in behavioral and educational interventions.
- **Power (1 − β)**: 0.80, indicating an 80% likelihood of detecting a true effect if one exists.
- **Significance level (α)**: 0.05, reflecting a 5% probability of Type I error.

**Fig. 1:**
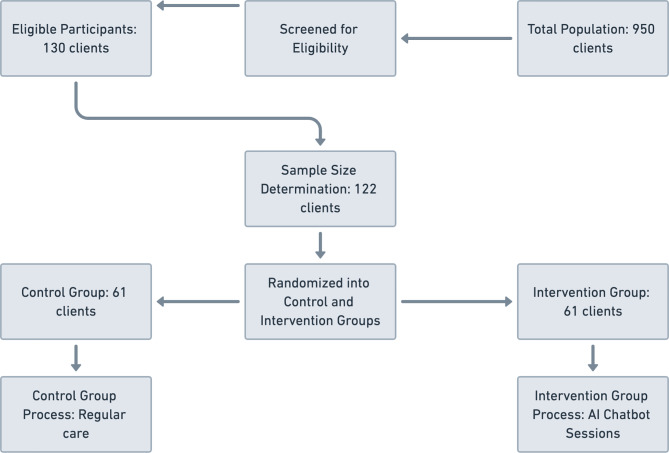
Allocation flowchart

These parameters resulted in a minimum required sample size of 110 participants. To account for potential attrition, the final recruitment target was set at 122 participants, ensuring the robustness of the findings (Fig. 1).

### Inclusion criteria

Participants had to satisfy the following criteria to be eligible for the study:

1. Female customers diagnosed with breast cancer, regardless of stage.
2. Participants must be 18 years or older to offer informed permission and participate in the intervention independently.
3. Ability to communicate with the AI chatbot and use learning tools requires Arabic literacy.
4. Willingness to participate in the research and interact with the chatbot during the intervention.

### Exclusion criteria

To protect the study’s integrity, the following exclusion criteria were used:

1. Clients with major cognitive impairments or serious mental health conditions may struggle to understand and engage in the session.
2. Patients receiving palliative care may choose symptom management above education.
3. Physically unable to attend group instruction sessions or evaluations.

### Recruitment and allocation to groups

Kafr El-Sheikh University Hospital, a reputable institution that serves a diverse population of breast cancer patients, conducted the recruitment process for this research over a four-week period at its oncology outpatient clinics. The recruitment method’s objective was to ensure that the selected participants accurately represented the target demographic and fulfilled the eligibility requirements necessary for effective engagement with the intervention.

The recruitment process commenced with the selection of potential volunteers by study assistants from the clinic’s attendance. The study’s objective was articulated plainly and succinctly, and breast cancer patients were actively involved during their routine follow-up appointments. The voluntary aspect of participation was emphasized to ensure that prospective participants felt comfortable with their involvement in the research. Individuals who expressed interest were assessed for eligibility in accordance with predetermined inclusion and exclusion criteria. Following the screening procedure, qualified individuals were provided with exhaustive information regarding the research, which encompassed its objectives, methodologies, potential benefits, and ethical safeguards. All participants provided written informed consent after receiving a full explanation of the study’s purpose, procedures, benefits, and risks. Participation was voluntary, and individuals were informed of their right to withdraw at any time without affecting their care. The participants were informed that their participation was entirely voluntary and that they could withdraw at any time without any repercussions on their current therapy.

The inclusion requirements were met by 130 of the 950 customers that were evaluated. In order to account for potential attrition and maintain statistical power, the research included 122 individuals, as determined by a specific sample size estimate.

In order to ensure an impartial allocation process, individuals were randomly assigned to two groups of equal size upon the conclusion of the recruitment process. A computer-generated sequence was used to randomize 61 individuals to the control group and the remaining 61 to the intervention group.

The control group received standard care, which included routine consultations with oncology specialists and nursing personnel at the outpatient clinics. This method established a baseline for comparison, as no additional educational or technological interventions were implemented for these participants.

In contrast, the intervention group participated in a structured program that included AI chatbot sessions in addition to standard care. The chatbot and its capabilities were introduced to the participants of this group during an orientation session. They received instructions on how to interact with the chatbot, which was intended to offer personalized educational content on breast cancer management, emotional support, and practical advice on treatment adherence and side effect management. The purpose of this digital aid was to enhance the in-person care they were already receiving, cultivate positive attitudes toward their condition and its management, and enhance their knowledge.

### Data collection tools

To thoroughly assess the effect of the AI chatbot intervention on breast cancer patients’ knowledge and attitudes, two meticulously designed and validated instruments were employed: the Artificial Intelligence of Breast Cancer Questionnaire (Tool I) and the Likert Scale for Attitude Towards AI (Tool II). These instruments were developed to obtain comprehensive, multidimensional data pertinent to the study’s aims.

### Tool I: artificial intelligence of breast Cancer questionnaire

This questionnaire was explicitly developed to evaluate the knowledge levels, perceptions of AI-based support, and empowerment of participants in the context of breast cancer management at the baseline and post-intervention stage. The instrument was composed of four primary sections, each of which was specifically designed to capture distinctive aspects of the participants’ experiences and comprehension.

1. **Demographic and Medical Data**:
  This section included items to collect basic demographic information (e.g., age, educational level, marital status) and clinical details (e.g., stage of breast cancer, duration of diagnosis, and treatment history). This data was essential for understanding the context of each participant’s responses and identifying patterns based on individual characteristics.
2. **Knowledge Assessment**:
  This section comprised 25 multiple-choice questions (MCQs) designed to assess participants’ knowledge of breast cancer, focusing on topics such as early detection, treatment options, risk factors, symptom management, and AI’s role in healthcare. Each correct answer was scored as **1 point**, while incorrect answers received **0 points**, resulting in a total score range of 0–25.
  **Scoring and Interpretation**: A participant’s knowledge was classified as:
    **Satisfactory**: ≥75% of the total score (≥ 19 points).
    **Unsatisfactory**: <75% of the total score (< 19 points).
      The knowledge assessment provided an objective measure of participants’ understanding, serving as a benchmark for evaluating the effectiveness of the chatbot intervention.
    Satisfactory knowledge was defined as achieving 75% or more of the total score (≥ 19 points out of 25), based on expert consensus and alignment with educational standards in health literacy assessment. This threshold was also supported by pilot testing with 15 breast cancer patients, which demonstrated that a score of 75% corresponded with correct understanding of core breast cancer and AI-related concepts. The cut-off aligns with prior studies using a similar scoring benchmark for competency in cancer education programs.
3. **AI Support and Empowerment**:
  This section aimed to measure participants’ perceptions of the chatbot’s role in enhancing their decision-making abilities and providing emotional empowerment. It included 10 Likert-scale questions, with responses ranging from 1 (Strongly Disagree) to 5 (Strongly Agree). Scores were aggregated to indicate overall perceptions, with higher scores reflecting greater perceived empowerment and support.

### Tool II: likert scale for attitude towards AI

This scale was developed to evaluate participants’ attitudes toward AI technology in healthcare, with a specific focus on its perceived utility, trustworthiness, and potential risks. It consisted of 24 carefully constructed statements across three domains:

1. **Trust in AI**:
  This domain included 8 statements assessing the participants’ trust in AI-based tools, such as their reliability, accuracy, and ability to provide unbiased information. Examples of statements included:
  “I trust AI to provide accurate and up-to-date information about breast cancer.”
  “AI can be a reliable tool for emotional support in healthcare.”
2. **Perceived Benefits of AI**:
  This domain included 8 statements focused on the perceived advantages of AI in enhancing healthcare delivery and patient support. Statements such as “AI can simplify complex medical information” and “AI can improve communication between patients and healthcare providers” reflected this aspect.
3. **Concerns About AI**:
  This sector included eight statments addressing possible objections and obstacles related to artificial intelligence usage, including privacy concerns, dependence on technology, and its incapacity to substitute human care. Among the statements were:
  “I am worried about the privacy of my personal information when using AI tools.”
  “AI lacks the emotional intelligence human healthcare providers offer.”

### Scoring and interpretation

scoring system provided a nuanced understanding of participants’ overall attitudes while allowing for domain-specific analyses. Every comment received a 5-point Likert scale score ranging from 1 (strongly disagree) to 5 (strongly agree). The overall score went from 24 to 120 and was understood as follows:

- Positive Attitude: 81–120 points.
- Neutral Attitude: 41–80 points.
- Negative Attitude: 0–40 points.

This scoring method enabled domain-specific studies and gave a complex knowledge of participants’ general opinions.

### Validation and reliability testing

To assure tools relevance, clarity, and suitability for the research population, both instruments were rigorously validated. Reviewing the materials for content validity, a team of five experts in cancer nursing, health informatics, and educational psychology examined Changes were done to guarantee that the instruments were culturally responsive to the backgrounds of the participants and fully addressed the goals of the research. Fifteen breast cancer clients—not part of the main study—were used for pilot testing under The results of this pilot phase helped us hone the questions, increase clarity, and guarantee that the tools were simple to use and complete. Cronbach’s alpha was the basis for dependability testing:

- Tool I: had an alpha value of 0.89, thereby demonstrating strong internal consistency for measuring of knowledge and empowerment.
- Tool II: obtained an alpha coefficient of 0.91, therefore demonstrating great consistency in opinions on artificial intelligence.

### Intervention development and chatbot design

The AI chatbot used in this study was developed in collaboration with a multidisciplinary team of oncology nurses, medical informaticians, and software engineers. Its content was informed by national breast cancer guidelines, WHO cancer education resources, and peer-reviewed literature on patient education and support. The chatbot was built using a rule-based natural language processing engine integrated with a curated database of breast cancer topics relevant to diagnosis, treatment, symptom management, and lifestyle modifications.

The training dataset comprised more than 200 structured queries drawn from clinical consultations, patient forums, and oncology FAQs. All content was reviewed and validated by two senior oncology nursing faculty and a clinical oncologist to ensure medical accuracy and cultural appropriateness. The chatbot underwent a three-phase validation process: content validation by experts, pilot testing with 15 breast cancer patients for usability, and technical debugging based on interaction logs.

Functionality included:

- Text-based conversational support accessible via mobile devices.
- Personalized education based on user input (e.g., treatment type, symptom queries).
- Pre-programmed responses for frequently asked emotional and practical concerns.
- Reassurance prompts and referral messages for critical queries.

User sessions were private, unlogged for identity, and data anonymized to maintain ethical integrity. While not generative AI (like ChatGPT), the chatbot followed a decision-tree model with layered content designed to guide patients step-by-step through their learning journey.

### Filed work

The fieldwork for this study was conducted at Kafr El-Sheikh University Hospital’s oncology outpatient clinics, where breast cancer patients receive comprehensive diagnostic and therapeutic care. Over a four-week period, participants were recruited based on predefined inclusion and exclusion criteria, resulting in the selection of 122 eligible patients out of 950 screened. These participants were then randomly assigned to either the intervention group (*n* = 61), which received AI chatbot-based education alongside standard oncology care, or the control group (*n* = 61), which continued with routine care alone. Prior to the intervention, all participants completed baseline assessments using the Artificial Intelligence of Breast Cancer Questionnaire and the Likert Scale for Attitude Towards AI, ensuring both groups had comparable knowledge and attitudes at the study’s start. The intervention group attended an orientation session, where they were introduced to the AI chatbot and trained on how to interact with it for personalized education, emotional support, and treatment guidance. Throughout the intervention period, participants in this group engaged with the chatbot beyond hospital hours, allowing for continuous access to tailored information. Chatbot usage metrics were monitored throughout the intervention using built-in analytics. These included the number of logins, total interaction time, and most frequently accessed content categories (e.g., treatment side effects, emotional support). On average, participants in the intervention group initiated 4.3 chatbot sessions (range 2–8), with a mean session duration of 12.6 min. Engagement was highest during evening hours, suggesting the chatbot was utilized outside traditional clinical access times. While individual message data were anonymized, cumulative interaction trends provided insight into content relevance and user interest.

### Data analysis

The collected data were analyzed using IBM SPSS Statistics (version 26), employing both descriptive and inferential statistical methods. Data were reviewed for completeness, coded, and tested for normality using the Shapiro-Wilk test. Descriptive statistics summarized demographic characteristics and baseline knowledge and attitude scores, with frequencies and percentages for categorical variables and means and standard deviations for continuous variables.

Inferential analysis included paired t-tests to compare pre- and post-intervention scores within the intervention group, and independent t-tests to compare post-intervention scores between the intervention and control groups. A chi-square test evaluated changes in the proportion of participants with satisfactory knowledge (≥ 75%). Effect sizes (Cohen’s d) quantified the magnitude of improvements, interpreted as small (d = 0.2), moderate (d = 0.5), or large (d = 0.8). Statistical significance was set at *P* <.05.

### Ethical considerations

This study adhered to the ethical standards announced in the Declaration of Helsinki, protecting participants’ autonomy, confidentiality, and wellbeing. Ethical permission was obtained from the Faculty of Nursing Ethics Committee at Kafr El-Sheikh University (IRB: KFSIRB200-383) in the Northen region of Egypt before to the establishing of the study. All participants were thoroughly apprised of the study’s objectives, methodologies, possible advantages, and dangers. the Informed concent was secured from each participant following comprehensive verbal and written explanations, assuring their comprehension of voluntary involvement and the opportunity to withdraw at any time without repercussions to their treatment. Confidentiality was maintained by assigning unique identities to participants and securely storing data in a password-protected system available only to the study team. Anonymity was maintained in the reporting of findings, so precluding the identification of individual individuals. Meticulous attention was devoted to preventing coercion during recruiting, and participants were informed that opting out would not affect their treatment. These ethical protocols protected participants’ rights and maintained the integrity of the study process.

## Results

Table 1 outlines the demographic and clinical characteristics of individuals in both the intervention and control groups, emphasizing their equivalence across essential factors. The average age of participants in the intervention group was somewhat lower (44.3 ± 7.8 years) than in the control group (45.7 ± 6.6 years), albeit this discrepancy was not statistically significant (*p* =.252). The distribution of marital status was comparable between the groups, with the majority of participants married (60.7% in the intervention group and 59.0% in the control group), followed by widowed/divorced and single individuals, revealing no significant differences across these categories (p-values > 0.05).The educational levels were evenly distributed among the groups, with a little greater percentage of participants in the intervention group having finished secondary school (47.5%) compared to the control group (42.6%). The percentage of individuals with university-level education or above was similar (27.9% vs. 29.5%; *p* =.836). The equilibrium in educational attainment is crucial as it minimizes the likelihood that any observed result discrepancies are affected by variations in initial educational levels. Concerning clinical features, the distribution of breast cancer stages was fairly uniform across groups, with Stage II being the predominant stage (41.0% in the intervention group and 44.3% in the control group), followed by Stages I and III. The same proportions in Stage III (29.5% in each group) further illustrate the equitable distribution of participation. The average period after diagnosis was somewhat greater in the intervention group (14.7 ± 4.3 months) than in the control group (13.4 ± 5.1 months), however this difference lacked statistical significance (*p* =.227).

**Table 1:** Demographic and clinical characteristics of the study participants (*N* = 122)

Variable	Intervention Group (*n* = 61)	Control Group (*n* = 61)	*p*-value
**Age (years)**,** M ± SD**	44.3 ± 7.8	45.7 ± 6.6	0.252
**Marital Status**, n (%)			
Single	11 (18.0)	14 (22.9)	0.397
Married	37 (60.7)	36 (59.0)	0.866
Widowed/Divorced	13 (21.3)	11 (18.0)	0.673
**Educational Level**, n (%)			
Primary/Elementary	15 (24.6)	17 (27.9)	0.657
Secondary	29 (47.5)	26 (42.6)	0.570
University/Above	17 (27.9)	18 (29.5)	0.836
**Breast Cancer Stage**, n (%)			
Stage I	18 (29.5)	16 (26.2)	0.668
Stage II	25 (41.0)	27 (44.3)	0.717
**Stage III**	18 (29.5)	18 (29.5)	1.00
**Mean Duration Since Diagnosis (months)**,** M ± SD**	14.7 ± 4.3	13.4 ± 5.1	0.227

The baseline knowledge ratings of participants in both the intervention and control groups prior to the AI chatbot intervention are illustrated in Table 2. The control group had a nearly identical mean score of 16.1 ± 2.8, while the intervention group had a mean knowledge score of 16.4 ± 2.3. This suggests that the groups had comparable levels of knowledge at the study’s inception, as the p-value of.351 does not indicate a statistically significant difference between them. When the results are categorized as satisfactory and unsatisfactory knowledge levels, they further substantiate the similarity between the categories. In the control group, 34.4% (*n* = 21) of participants demonstrated a satisfactory level of knowledge (≥ 19 points), while 36.1% (*n* = 22) of participants in the intervention group did so. Conversely, the majority of participants in both groups had insufficient knowledge scores, with 63.9% (*n* = 39) in the intervention group and 65.6% (*n* = 40) in the control group each scoring below the 75% threshold. The p-value of.849 for knowledge classification confirms that the differences between the two groups were not statistically significant.

**Table 2:** Baseline knowledge score on breast Cancer and AI (*N* = 122)

Knowledge Score (0–25)	Intervention Group (*n* = 61)	Control Group (*n* = 61)	*p*-value
**Mean ± SD**	16.4 ± 2.3	16.1 ± 2.8	0.351
**Knowledge Classification**, n (%)			
Satisfactory (≥ 19)	22 (36.1)	21 (34.4)	0.849
Unsatisfactory (< 19)	39 (63.9)	40 (65.6)	0.849

Table 3 displays the post-intervention knowledge scores of individuals from both the intervention and control groups, organized by essential knowledge areas. The findings indicate a significant improvement in knowledge among participants in the intervention group relative to the control group, especially in AI-specific knowledge and overall knowledge scores. In the Basic Concepts domain (maximum score: 8), the intervention group attained a mean score of 6.1 ± 1.2, while the control group recorded a score of 5.0 ± 1.8. The mean difference of 1.1 (95% CI: 0.7 to 1.5, *p* =.002) indicates a statistically significant improvement in core breast cancer knowledge among those exposed to the AI chatbot. The effect size (Cohen’s d = 0.67) indicates a moderate to substantial influence. In the Treatment & Side Effects domain (maximum score: 7), the intervention group achieved a score of 4.9 ± 1.3, while the control group scored 4.3 ± 1.4. The mean difference of 0.6 (95% CI: 0.2 to 1.0, *p* =.012) indicates a small although statistically significant enhancement, with an effect size (Cohen’s d = 0.43) reflecting a moderate influence. The greatest significant improvement was seen in the AI-Specific Knowledge category (highest score: 10). The intervention group achieved a considerably higher score (8.9 ± 2.2) compared to the control group (6.2 ± 2.5), resulting in a mean difference of 2.7 (95% CI: 2.0 to 3.4, *p* <.001). The substantial effect size (Cohen’s d = 1.15) underscores the significant influence of the AI chatbot on improving participants’ comprehension of AI applications in breast cancer treatment. The overall knowledge score (out of 25) was much greater in the intervention group (20.3 ± 2.1) than in the control group (17.9 ± 3.4), exhibiting a mean difference of 2.4 (95% CI: 1.7 to 3.1, *p* <.001) and a considerable effect size (Cohen’s d = 0.82). This underscores the efficacy of the AI chatbot in enhancing users’ knowledge completely. The percentage of participants possessing good knowledge (≥ 19 points) was significantly greater in the intervention group (78.7%) compared to the control group (41.0%) (*p* <.001).

**Table 3:** Post-Intervention knowledge scores by Sub-Domain (*N* = 122)

Knowledge Domain	Intervention (*n* = 61)	Control (*n* = 61)	Mean Diff (95% CI)	*p*-value	Cohen’s d
A. Basic Concepts (0–8)	6.1 ± 1.2	5.0 ± 1.8	1.1 (0.7 to 1.5)	0.002	0.67
B. Treatment & Side Effects (0–7)	4.9 ± 1.3	4.3 ± 1.4	0.6 (0.2 to 1.0)	0.012	0.43
C. AI-Specific Knowledge (0–10)	8.9 ± 2.2	6.2 ± 2.5	2.7 (2.0 to 3.4)	< 0.001	1.15
Overall Knowledge (0–25)	20.3 ± 2.1	17.9 ± 3.4	2.4 (1.7 to 3.1)	< 0.001	0.82
Satisfactory Knowledge (≥ 19), n (%)	48 (78.7)	25 (41.0)	—	< 0.001	—

Table 4 shows substantial improvements in knowledge across all categories within the intervention group subsequent to interaction with the AI chatbot. The Basic Concepts domain exhibited a significant rise from 4.7 ± 1.4 at baseline to 6.1 ± 1.2 post-intervention, with a mean change of 1.4 (95% CI: 1.0 to 1.8, *p* <.001, d = 1.00), indicating a substantial impact size. Correspondingly, knowledge about Treatment & Side Effects shown a substantial enhancement from 3.6 ± 1.3 to 4.9 ± 1.3, resulting in a mean change of 1.3 (95% CI: 0.9 to 1.7, *p* <.001, d = 0.94), indicating a significant educational effect. The greatest significant enhancement was seen in AI-Specific Knowledge, with the mean score rising from 6.2 ± 2.1 to 8.9 ± 2.2, resulting in a mean change of 2.7 (95% CI: 2.1 to 3.3, *p* <.001, d = 1.21), hence substantiating the chatbot’s efficacy in improving AI-related awareness. The overall knowledge score increased from 16.4 ± 2.3 to 20.3 ± 2.1, with a mean change of 3.9 (95% CI: 3.2 to 4.6, *p* <.001, d = 1.06), indicating a substantial and significant effect of the intervention. The results validate the AI chatbot’s efficacy in substantially improving breast cancer knowledge, especially on AI-related topics, with all effect sizes above 0.9, indicating a robust educational advantage.

**Table 4:** Within-Group changes in knowledge scores for the intervention arm (*n* = 61)

Knowledge Domain	Baseline	Post-Intervention	Mean Change (95% CI)	*p*-value	Cohen’s d
A. Basic Concepts (0–8)	4.7 ± 1.4	6.1 ± 1.2	1.4 (1.0 to 1.8)	< 0.001	1.00 (large)
B.Treatment & Side Effects (0–7)	3.6 ± 1.3	4.9 ± 1.3	1.3 (0.9 to 1.7)	< 0.001	0.94 (large)
C.AI-Specific Knowledge (0–10)	6.2 ± 2.1	8.9 ± 2.2	2.7 (2.1 to 3.3)	< 0.001	1.21 (large)
Overall Knowledge (0–25)	16.4 ± 2.3	20.3 ± 2.1	3.9 (3.2 to 4.6)	< 0.001	1.06 (large)

Table 5 demonstrates a significantly more positive attitude towards AI among participants in the intervention group compared to the control group. The intervention group exhibited higher trust in AI (31.4 ± 4.1 vs. 26.8 ± 5.3, *p* <.001, d = 0.92) and greater perceived benefits of AI (32.7 ± 3.8 vs. 27.1 ± 4.9, *p* <.001, d = 1.02), indicating increased confidence in AI as a reliable healthcare tool. Notably, concerns about AI were lower in the intervention group (18.3 ± 5.6 vs. 22.7 ± 6.4, *p* <.001, d = 0.87), suggesting that chatbot interaction helped alleviate fears related to privacy, reliability, and emotional engagement. The overall attitude score (82.4 ± 7.2 vs. 72.6 ± 8.9, *p* <.001, d = 1.15) further confirms a substantial improvement in AI acceptance, reinforcing the chatbot’s role in shaping positive perceptions and fostering greater AI adoption in healthcare settings.

**Table 5:** Post-Intervention attitude towards AI in healthcare (*N* = 122)

Attitude Domain	Intervention (*n* = 61)	Control (*n* = 61)	Mean Difference (95% CI)	*p*-value	Cohen’s d
Trust in AI (0–40)	31.4 ± 4.1	26.8 ± 5.3	4.6 (3.2 to 6.0)	< 0.001	0.92 (large)
Perceived Benefits (0–40)	32.7 ± 3.8	27.1 ± 4.9	5.6 (4.1 to 7.1)	< 0.001	1.02 (large)
Concerns About AI (0–40)	18.3 ± 5.6	22.7 ± 6.4	-4.4 (-5.9 to -2.9)	< 0.001	0.87 (large)
Overall Attitude Score (0–120)	82.4 ± 7.2	72.6 ± 8.9	9.8 (7.3 to 12.3)	< 0.001	1.15 (large)

Table 6 displays the logistic regression analysis that identifies characteristics correlated with a favorable attitude toward AI among breast cancer patients. The adjusted odds ratios (AOR) and 95% confidence intervals (CI) reflect the probability of a person forming a favorable view of AI, contingent upon significant demographic and clinical factors. Age did not significantly influence attitudes toward AI (AOR = 1.02, 95% CI: 0.97–1.06, *p* =.316), indicating that opinions of AI were uniform across all age demographics. Education level significantly influenced outcomes, as those with secondary education were 1.34 times more likely to have a favorable attitude towards AI than those with just primary school (95% CI: 1.02–1.62, *p* =.041). The impact was more pronounced in those with a university degree or above, who exhibited a 1.57-fold increased probability of a positive attitude (95% CI: 1.10–2.19, *p* =.018). The data indicate that more education is associated with increased acceptance and confidence in AI. The stage of breast cancer did not markedly affect views about AI, as neither Stage II compared to Stage I (AOR = 1.09, *p* =.587) nor Stage III compared to Stage I (AOR = 1.16, *p* =.374) demonstrated significant relationships. This suggests that the severity of the condition does not significantly influence opinions regarding AI-based therapies. The primary predictor of a favorable attitude was knowledge acquisition, where each unit increase in post-intervention knowledge scores correlated with a 1.73-fold increase in the probability of a positive AI attitude (95% CI: 1.24–2.12, *p* =.002).

**Table 6:** Logistic regression analysis for predictors of positive attitude toward AI (*N* = 122)

Variable	AOR	95% CI	*p*-value
Age (years)	1.02	0.97–1.06	0.316
Education Level			
Secondary vs. Primary/Elementary	1.34	1.02–1.62	0.041
University/Above vs. Primary/Elem.	1.57	1.10–2.19	0.018
Breast Cancer Stage			
Stage II vs. Stage I	1.09	0.76–1.45	0.587
Stage III vs. Stage I	1.16	0.85–1.58	0.374
Knowledge Gains (score difference)	1.73	1.24–2.12	0.002

**Fig. 2:**
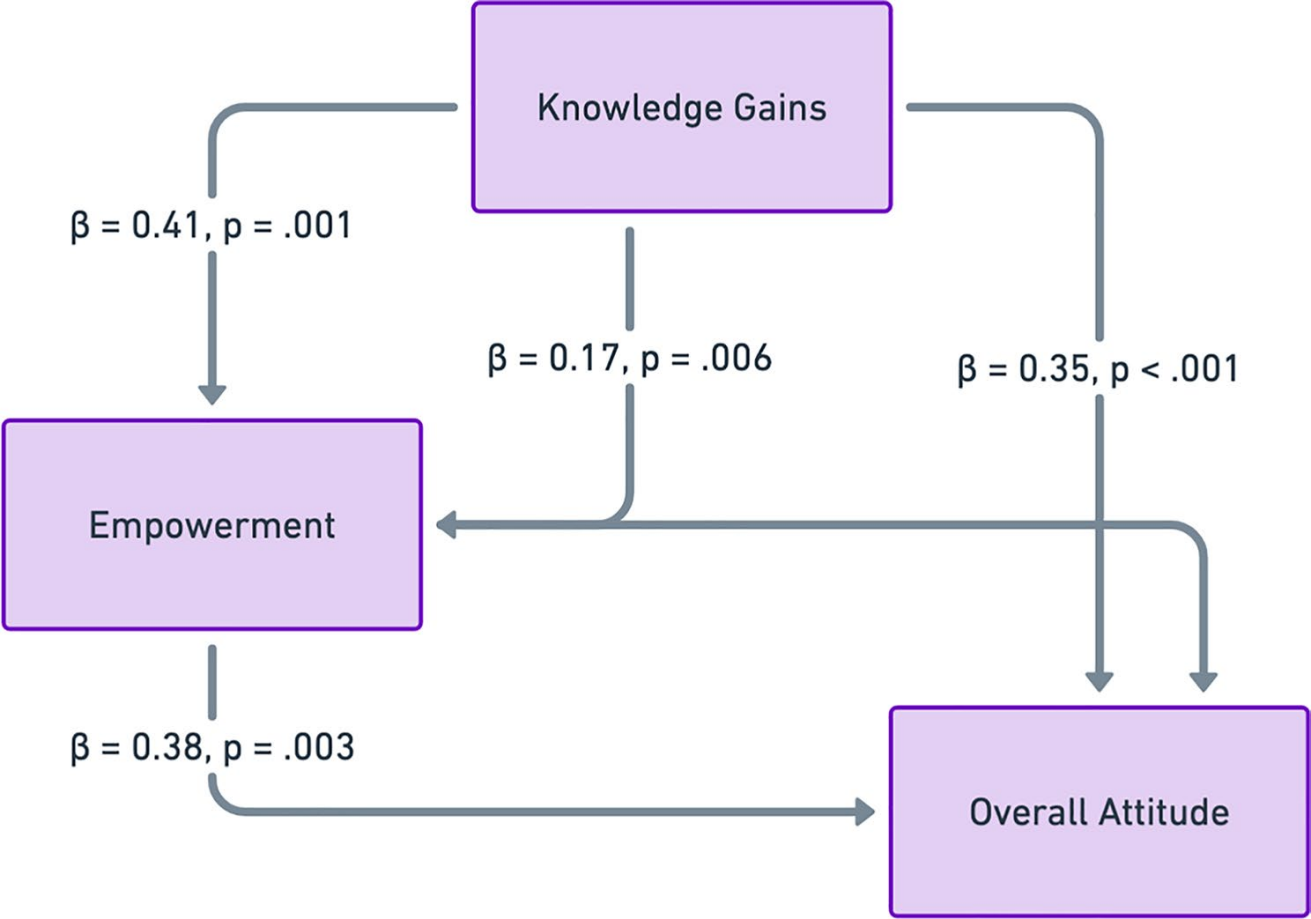
Pathway analysis model

Table 7; Fig. 2 displays the results of a path analysis model investigating the direct and indirect associations among knowledge gains, empowerment, and general attitudes toward AI within the intervention group. The results underscore that enhancements in knowledge foster participants’ feeling of empowerment and consequently influence their perceptions of AI in healthcare.Knowledge increases substantially predicted empowerment (β = 0.41, *p* =.001, 95% CI: 0.19–0.62), suggesting that enhanced knowledge resulted in higher confidence and autonomy in decision-making. Empowerment significantly enhanced the overall attitude toward AI (β = 0.38, *p* =.003, 95% CI: 0.14–0.53), indicating that persons who experienced more empowerment were more inclined to see AI positively. Moreover, knowledge acquisition significantly affected overall attitude (β = 0.35, *p* <.001, 95% CI: 0.17–0.49), indicating that an improved comprehension of AI’s function in breast cancer treatment fostered a more favorable view of AI-driven therapies. The indirect influence of information acquisition on overall attitude, mediated by empowerment, was statistically significant (β = 0.17, *p* =.006, 95% CI: 0.05–0.26). This finding emphasizes that empowerment acts as an essential link between knowledge acquisition and AI acceptance, indicating that participants who acquired greater knowledge not only enhanced their understanding of AI but also experienced increased control over their healthcare decisions, thereby strengthening their favorable disposition towards AI.

**Table 7:** Path analysis model: direct and indirect effects on overall attitude (Intervention group, *n* = 61)

Path	Standardized β	*p*-value	95% CI
**Direct Effects**			
Knowledge Gains → Empowerment	0.41	0.001	0.19–0.62
Empowerment → Overall Attitude	0.38	0.003	0.14–0.53
Knowledge Gains → Overall Attitude	0.35	< 0.001	0.17–0.49
**Indirect Effect** (Mediation)			
Knowledge Gains → Empowerment → Overall Attitude	0.17	0.006	0.05–0.26

Table 8 Highlights the significant impact of the AI chatbot on participants’ perceived support and empowerment. The intervention group reported markedly higher scores in decision-making support (8.2 ± 1.5 vs. 6.3 ± 1.9, *p* <.001, d = 0.92) and emotional support (7.9 ± 1.8 vs. 5.8 ± 2.2, *p* <.001, d = 1.02) compared to the control group, indicating that chatbot interaction facilitated both informational and psychological support. Additionally, the overall empowerment score (41.5 ± 4.3 vs. 35.2 ± 5.6, *p* <.001, d = 1.15) was significantly higher in the intervention group, demonstrating the chatbot’s effectiveness in enhancing patients’ confidence in managing their condition.

**Table 8:** Post-Intervention AI support and empowerment scores (*N* = 122)

Empowerment Domain	Intervention (*n* = 61)	Control (*n* = 61)	Mean Difference (95% CI)	*p*-value	Cohen’s d
Decision-Making Support (0–10)	8.2 ± 1.5	6.3 ± 1.9	1.9 (1.3 to 2.5)	< 0.001	0.92 (large)
Emotional Support (0–10)	7.9 ± 1.8	5.8 ± 2.2	2.1 (1.4 to 2.8)	< 0.001	1.02 (large)
Overall Empowerment (0–50)	41.5 ± 4.3	35.2 ± 5.6	6.3 (4.8 to 7.8)	< 0.001	1.15 (large)

## Discussion

World Health Organization estimates report that breast cancer is the leading cause of cancer-related mortality among females and a major contributor to the overall cancer burden in 2022, ranking as the second most diagnosed cancer in 2022 [30]. The persistently elevated incidence rates impose a significant burden on health systems, especially in low-middle-income countries where early detection, diagnosis, and management remain challenging [31]. Integrating Innovative digital technologies, including artificial intelligence-powered chatbots, can provide promising solutions by increasing patients’ knowledge and awareness about symptoms, treatment options and side effects, and lifestyle modifications [32]. ChatGPT is an example of a generative pre-trained transformer that is openly accessible online. To the best of our knowledge, this is one of the few studies evaluating the impact of AI-powered chatbots on breast cancer patients’ knowledge, empowerment, and attitudes [33].

The inclusion of patients across all breast cancer stages was intentional to reflect the diversity of real-world clinical populations. While disease stage can influence informational and emotional needs, the chatbot was designed with modular content tailored to varying points along the cancer continuum. For example, early-stage patients accessed information on diagnosis and surgical options, while those undergoing treatment were guided on chemotherapy side effects and coping strategies. Emotional support components and frequently asked questions were universally available and written at varying literacy levels. Nonetheless, we acknowledge that certain psychosocial or symptom-related challenges may require further personalization. Future enhancements could incorporate stage-specific pathways within the chatbot algorithm or integrate adaptive learning features that adjust content based on user interactions over time.

This study demonstrated that integrating a structured AI chatbot intervention into oncology care significantly enhanced breast cancer patients’ knowledge, attitudes, and perceived empowerment—core outcomes that directly align with the study’s aim to evaluate educational and attitudinal change through AI-driven support. The findings of this study demonstrated significant improvements in breast cancer patients’ knowledge, attitudes, and perceived empowerment following the use of an AI-powered chatbot. Given the observed gains in AI-specific knowledge and trust, this study supports the potential of chatbots to serve as scalable, nurse-augmented digital tools in health education strategies—particularly for complex conditions like breast cancer., especially in increasing engagement, knowledge acquisition, and fostering positive attitudes toward digital health. This study underscores the importance of integrating AI-driven tools like AI-powered chatbots into patient education and support strategies, particularly in oncology nursing [34].

Understanding the quality of patient health information and the way in which it is accessed is vital in the digital information age. Our findings revealed a statistically significant difference in participants’ knowledge scores between the intervention and control group after the implementation of an AI-powered chatbot intervention. Participants’ overall knowledge scores improved significantly after the intervention, particularly AI-specific knowledge. Compared to the control group, participants in the intervention group demonstrated significantly higher knowledge scores in post-intervention, emphasizing that knowledge acquisition through chatbot-based education is an effective method for increasing knowledge of breast cancer. Moreover, there was a substantial positive impact of integrating AI-driven educational interventions into nursing care for breast cancer patients, as the proportion of participants who achieved a satisfactory level of knowledge was significantly greater in the intervention group (78.7%) than in the control group (41.0%), highlighting the substantial positive impact of integrating AI-driven educational interventions into nursing care for breast cancer patients [35].

Our study results align with study investigated the capability of AI conversational agents called Vik was able to offer information to patients with breast cancer about breast cancer and its epidemiology, treatments, side effects, and quality of life improvement strategies (sport, fertility, sexuality, and diet) [36]. This study showed that AI chatbots could provide information and satisfactory answers to breast cancer women and the scores from the chatbot were found to be non-inferior to the scores of the group of physicians, confirming the chatbot’s effectiveness in delivering patient-centred informational support. Similarly, study, examined the effect of smartphone-based artificial intelligence chatbots on older adults with cancer and reported significant improvements in patient engagement, adherence to treatment regimes, and structured monitoring of symptoms [37].

Moreover, Chaix et al., utilized a chatbot to address questions regarding breast cancer and reinforce medication adherence by providing prescription reminders throughout the conversation to reinforce medication adherence [12]. As a result, 88% of patients reported that the chatbot provided them with support and enabled them to effectively follow their treatment regimen. According to the above-mentioned studies, AI-driven conversational agents could enhance empowerment through continuous access to personalized information, satisfaction, and support of cancer patients and increased compliance with treatment plans as well.

Concerning participants’ attitudes toward AI-driven chatbots in healthcare, the intervention group appeared to have a significant positive attitude toward AI-driven chatbots in the three main domains: Trust in AI, Perceived benefits of AI and Concern about AI compared to the control group. The participants reported significantly higher scores in Trust in AI and greater Perceived benefits of AI. Additionally, the overall attitudinal score again underlines a positive role to be played by the chatbot in positively influencing perceptions and acceptability of AI. Based on this, AI chatbot interventions are purported to support and increase women’s acceptance while promoting positive attitudes toward integrating advanced digital technologies into oncology nursing care [38].

Our study results are consistent with several studies using AI-driven chatbots in oncology care. Tawfik et al., a randomized controlled trial study investigated the effects of an AI-powered chatbot (ChemoFreeBot) compared to nurse-led education on the effectiveness of self-care behaviours and the distress of chemotherapy side effects among breast cancer patients [23]. This study revealed that Women who engaged with the ChemoFreeBot had the most effective self-care behaviours and the lowest psychological symptom frequency, severity and distress postintervention. It was highlighted that ChemoFreeBot offered women living with breast cancer an effective and cost-effective tool to help them improve self-care behavior and reduce chemotherapy side effects by providing tailored educational programs and improving access to quality information [39].

Another study conducted by Yang et al., to evaluate the attitudes of Chinese cancer patients toward the clinical use of artificial intelligence and to analyze the possible influencing factors, revealed that most cancer patients believed in AI and had a positive attitude toward using AI [40]. Notably similar to our research is the study by Yusuf et al.,, to assess the efficacy of BrAware chatbot Apps in enhancing the knowledge of breast cancer screening, awareness of warning signs and confidence in breast self-examination (BSE), found that BrAware chatbot app can be considered feasible in a real clinical context to promote behavioural changes in the lifestyles of women in performing Breast cancer screening [41]. One possible explanation is that the app encourages repeated practice and engagement of health-related skills, which may enhance memory retention and reinforce positive health attitudes.

Despite the potential benefits of chatbots in providing positive attitudes and facilitating emotional support for cancer patients, the finding of Goumas et al.,, highlighted critical challenges such as chatbots may produce coherent sounding but erroneous or inaccurate information, a phenomenon known as “hallucinations” because they predict answers instead of understanding the meaning of those responses [11]. Moreover, there are concerns regarding data privacy, digital exclusion as well as chatbots’ inability to understand human emotions, and situations when medical expertise and intelligence are required particularly in oncology care [42].

The multivariate logistic regression revealed a significant positive relationship between participants’ attitudes toward AI and their educational level. It seems plausible that Education level significantly influenced outcomes, as participants with higher education levels are more likely to perceive AI positively. These findings suggest that the educational level is positively associated with greater acceptance and confidence in AI-driven chatbots. This may be attributed to their increased familiarity with technology and greater confidence in accessing and utilizing digital health resources. Consequently, this familiarity reduced fear of the unknown and helped build trust through positive experiences. As Hilbers et al. point out, that patient familiarity with AI technology is a key factor in shaping patient acceptance and trust [43].

These findings align with the literature suggesting that greater AI knowledge leads to more favorable attitudes towards the technology. Therefore, the role of levels of education on the attitude toward AI was supported by evidence from Fritsch al., found that higher-educated patients had more to have heard about and became conversant with artificial intelligence in medicine, which was indirectly associated with more favourable acceptance and use [44]. Nonetheless, the existence of a minority that expresses disagreement or strong disagreement is noteworthy and it came as no surprise, the findings of Hamedani et al.,, study revealed that there is no statistically significant relationship between the level of education and attitude toward AI. As a result of this divergence, it is evident that there is skepticism or resistance towards cognitive technology in healthcare, underscoring the need for careful consideration and management of AI implementation [45].

The path analysis model suggested plausible associations, indicating that knowledge gain and empowerment may contribute to attitudinal changes among participants who received the AI-powered chatbot. These findings highlight potential pathways, but due to the observational nature of path models, they should not be interpreted as definitive causal relationships. In the study, knowledge gain showed the strongest direct effect on empowerment, suggesting that participants could become more autonomous in their decision-making process and perceive themselves as being more capable as a result of increased knowledge. Similarly, empowerment directly affected overall attitude, underscoring its significant role in shaping positive perceptions toward attitude change. Additionally, we found a significant direct path between knowledge gains and overall attitude, suggesting that knowledge alone may contribute to changes in women’s attitudes through increased awareness [46].

According to Tribble et al.,, stated that knowledge gain is a crucial component of empowerment, and empowerment reinforces a variety of behaviours as well as encourages individuals to take more control of their lives. The learning experience is facilitated, in which individuals can try new approaches, change attitudes, and apply real-life learning, highlighting the significance of knowledge for empowerment as a whole [47]. Furthermore, a recent systematic review and meta-analysis study conducted by Kuo et al., demonstrated that knowledge gain was consistently associated with increased empowerment, regardless of the format or context of the intervention [48]. A possible explanation is that empowerment manifests itself in the attitude of a patient through conscious control over behaviour and self-efficacy, which allows them to make rational decisions.

Conversely, Taylor et al., found that people with greater knowledge of data use tend to hold more negative attitudes toward them, contradicting the relationship between knowledge and attitude found in our study. Probably, this could be explained by discrepancies in the nature and context of the knowledge being transferred [49].

Our findings are supported by Vainauskienė, who stated that empowerment enables the patients with power and trust by increasing their awareness and knowledge empowerment refers to the ability of the participants to use what they have learned to promote healthy behaviours and positively shape their attitudes through their everyday decision-making processes [50]. Similarly, Cardoso et al., demonstrated that empowerment interventions can enhance patient autonomy in shared decision-making by helping patients develop coping skills, communication skills, and behavioural changes related to their disease [51].

Although there is substantial evidence supporting the mediation relationship, some studies contradict our findings. Pruijt and Yerkes, assert that knowledge, empowerment, and attitudes are not always positively correlated [52]. Their study results indicate that empowerment can occasionally create tension between autonomy and control, potentially counteracting the positive attitudinal changes that might arise from knowledge gain. The disparities may be attributed to the nature of the information given, and its relevance to participants’ needs, which may have augmented the efficacy of this mediation process.

According to our findings, compared to the control group, the participants in the AI chatbot intervention group experienced significant improvements regarding emotional support with statistical differences, suggesting that AI chatbot interactions have effectively enhanced participants’ perceptions of emotional support in managing their health conditions. The results of our study are comparable with the findings of Hasei et al.,, which found that generative AI chatbots empower patients through emotional support, suggesting that AI chatbots are effective in addressing psychological concerns in healthcare [53]. Despite these positive findings, Kurniawan et al., mentioned several complex medical concerns that cannot be adequately addressed by artificial intelligence chatbots and still require human intervention to be successful and emphasized that chatbots cannot replace human interaction in the pursuit of patient-empathetic care [54].

Overall, the findings of our study emphasize that artificial intelligence-powered chatbots can play crucial a role in augmenting breast cancer patients’ empowerment by enhancing their knowledge about symptoms, treatment options and adherence, side effects, lifestyle change as well as their attitudes. Consequently, AI-driven chatbots have a great potential to positively impact oncology nursing care, especially among breast cancer patients.

### Limitations

This study was conducted at a single university hospital, which may limit the generalizability of the findings to broader or more diverse populations. While the sample was demographically and clinically heterogeneous, it may not fully represent breast cancer patients in rural, low-resource, or non-academic settings. Additionally, while attrition was minimal and accounted for in the sample size calculation, no imputation techniques were used for missing data due to the high response rate. Future multicenter trials with more diverse populations and longer-term follow-up are recommended to strengthen external validity and assess the sustainability of chatbot-driven improvements.

While the findings demonstrate substantial improvements in knowledge and attitudes among the intervention group, the possibility of the Hawthorne effect cannot be ruled out. Participants in the intervention group received additional engagement through chatbot orientation and follow-up, which may have influenced their motivation or attentiveness during assessments. Moreover, the large effect sizes observed—particularly in AI-specific knowledge (Cohen’s d > 1)—raise the possibility of measurement bias or social-desirability effects, where participants may respond more favorably due to perceived expectations. Future studies should consider blinding outcome assessments, incorporating objective engagement metrics, and including follow-up assessments to evaluate the durability of intervention effects and minimize potential bias.

Despite the promising results, limitations related to AI chatbot functionality must be acknowledged. One notable concern is the potential for “hallucinations”—instances where chatbots generate plausible-sounding but incorrect or misleading responses. This issue, documented by Goumas et al. [11], can compromise the accuracy of health information and erode trust. While our chatbot was trained on verified content and reviewed by domain experts, the inherent unpredictability of generative AI models necessitates safeguards. Future iterations should incorporate rigorous content validation protocols, user feedback mechanisms, and AI explainability tools to reduce misinformation risks. Additionally, digital exclusion and the chatbot’s inability to interpret nuanced emotional cues remain limitations, especially in sensitive oncology settings.

## Conclusion

This study demonstrated that integrating an AI chatbot into the standard care of breast cancer patients can substantially enhance their knowledge and foster more positive attitudes toward AI in healthcare. Participants who engaged with the chatbot showed significant improvements in overall understanding of breast cancer concepts and AI-specific information, compared to those who received standard care alone. Logistic regression findings underscored that both higher educational level and greater knowledge gains strongly predicted a favorable attitude toward AI, highlighting the importance of targeted, evidence-based educational tools.

Moreover, the path analysis shed light on how knowledge gains operated both directly and indirectly, via increased empowerment, to bolster participants’ acceptance of AI in their treatment journey. These mediating effects suggest that AI-driven interventions not only deliver factual information but also encourage a sense of control and self-efficacy among breast cancer patients. By bridging information gaps and offering timely emotional support, the chatbot intervention addressed a key limitation in the traditional cancer-care model, namely, inconsistent patient education and limited after-hours support.

In practice, these findings emphasize the need for interdisciplinary collaboration in oncology nursing, informatics, and psychosocial support to develop and implement user-friendly, culturally competent AI chatbot solutions. Future research might explore ways to scale up such interventions across diverse healthcare settings and investigate long-term patient outcomes, including adherence to treatment regimens, quality of life, and clinical markers. Overall, adopting AI chatbots as part of a comprehensive cancer-care strategy holds promise for improving patient engagement, empowerment, and health-related decision-making.

## Electronic supplementary material

Below is the link to the electronic supplementary material.

Supplementary Material 1

## Data Availability

The datasets generated during and/or analyzed during the current study are available from the corresponding author on reasonable request.
