# Supplementary material for: Empowering breast cancer clients through AI chatbots: transforming knowledge and attitudes for enhanced nursing care
Source: BMC Nurs. 2025 Jul 29;24:994. doi: 10.1186/s12912-025-03585-w (PMC12309207; doi:10.1186/s12912-025-03585-w)
Supplement: Supplementary file 1 — Supplementary Material 1 [file 12912_2025_3585_MOESM1_ESM.docx]

# Artificial Intelligence of Breast Cancer Questionnaire

This questionnaire has been developed to assess the knowledge, attitudes, and empowerment of breast cancer patients in relation to AI-powered healthcare technologies. Your responses will contribute to the understanding and development of supportive digital interventions. Please answer all items to the best of your ability.

## Tool I: Artificial Intelligence of Breast Cancer Questionnaire

### Section 1: Demographic and Medical Data

- 1. Age: ___________
- 2. Marital Status: [ ] Single [ ] Married [ ] Divorced/Widowed
- 3. Educational Level: [ ] Primary [ ] Secondary [ ] University or above
- 4. Stage of Breast Cancer: [ ] I [ ] II [ ] III
- 5. Duration since diagnosis (months): ___________
- 6. Treatment history (please specify): _________________________________

### Section 2: Knowledge Assessment (Choose the correct answer)

1. 1. [Sample Question Placeholder]
    a) Option A
    b) Option B
    c) Option C
    d) Option D
2. 2. [Sample Question Placeholder]
    a) Option A
    b) Option B
    c) Option C
    d) Option D
3. 3. [Sample Question Placeholder]
    a) Option A
    b) Option B
    c) Option C
    d) Option D
4. 4. [Sample Question Placeholder]
    a) Option A
    b) Option B
    c) Option C
    d) Option D
5. 5. [Sample Question Placeholder]
    a) Option A
    b) Option B
    c) Option C
    d) Option D
6. 6. [Sample Question Placeholder]
    a) Option A
    b) Option B
    c) Option C
    d) Option D
7. 7. [Sample Question Placeholder]
    a) Option A
    b) Option B
    c) Option C
    d) Option D
8. 8. [Sample Question Placeholder]
    a) Option A
    b) Option B
    c) Option C
    d) Option D
9. 9. [Sample Question Placeholder]
    a) Option A
    b) Option B
    c) Option C
    d) Option D
10. 10. [Sample Question Placeholder]
     a) Option A
     b) Option B
     c) Option C
     d) Option D
11. 11. [Sample Question Placeholder]
     a) Option A
     b) Option B
     c) Option C
     d) Option D
12. 12. [Sample Question Placeholder]
     a) Option A
     b) Option B
     c) Option C
     d) Option D
13. 13. [Sample Question Placeholder]
     a) Option A
     b) Option B
     c) Option C
     d) Option D
14. 14. [Sample Question Placeholder]
     a) Option A
     b) Option B
     c) Option C
     d) Option D
15. 15. [Sample Question Placeholder]
     a) Option A
     b) Option B
     c) Option C
     d) Option D
16. 16. [Sample Question Placeholder]
     a) Option A
     b) Option B
     c) Option C
     d) Option D
17. 17. [Sample Question Placeholder]
     a) Option A
     b) Option B
     c) Option C
     d) Option D
18. 18. [Sample Question Placeholder]
     a) Option A
     b) Option B
     c) Option C
     d) Option D
19. 19. [Sample Question Placeholder]
     a) Option A
     b) Option B
     c) Option C
     d) Option D
20. 20. [Sample Question Placeholder]
     a) Option A
     b) Option B
     c) Option C
     d) Option D
21. 21. [Sample Question Placeholder]
     a) Option A
     b) Option B
     c) Option C
     d) Option D
22. 22. [Sample Question Placeholder]
     a) Option A
     b) Option B
     c) Option C
     d) Option D
23. 23. [Sample Question Placeholder]
     a) Option A
     b) Option B
     c) Option C
     d) Option D
24. 24. [Sample Question Placeholder]
     a) Option A
     b) Option B
     c) Option C
     d) Option D
25. 25. [Sample Question Placeholder]
     a) Option A
     b) Option B
     c) Option C
     d) Option D

Scoring: Each correct answer = 1 point; incorrect = 0 points. Total score = 0–25.
Interpretation:
• Satisfactory: ≥19 points
• Unsatisfactory: <19 points

### Section 3: AI Support and Empowerment (1=Strongly Disagree to 5=Strongly Agree)

1. 1. The chatbot helped me understand my treatment options.
2. 2. I felt supported by the chatbot during my health journey.
3. 3. The chatbot helped me feel more in control of my decisions.
4. 4. I received emotional support from the chatbot.
5. 5. I found the chatbot easy to interact with.
6. 6. The chatbot responses were timely and relevant.
7. 7. The chatbot enhanced my confidence in managing breast cancer.
8. 8. I would recommend the chatbot to others.
9. 9. The chatbot provided useful advice for dealing with side effects.
10. 10. I felt empowered by using the chatbot.

## Tool II: Likert Scale for Attitude Towards AI (1=Strongly Disagree to 5=Strongly Agree)

### Domain 1: Trust in AI

1. 1. I trust AI to provide accurate and up-to-date information about breast cancer.
2. 2. AI can be a reliable tool for emotional support in healthcare.
3. 3. I believe AI systems are unbiased in delivering healthcare information.
4. 4. AI can assist doctors in making better treatment decisions.
5. 5. AI technology works reliably without frequent errors.
6. 6. AI recommendations are based on scientific evidence.
7. 7. I believe AI is safe to use in healthcare.
8. 8. AI can maintain confidentiality of patient information.

### Domain 2: Perceived Benefits of AI

1. 1. AI simplifies complex medical information.
2. 2. AI can provide 24/7 access to health information.
3. 3. AI can reduce the burden on healthcare professionals.
4. 4. AI improves communication between patients and healthcare providers.
5. 5. AI helps me to manage my health better.
6. 6. AI provides personalized health recommendations.
7. 7. AI saves time in receiving health advice.
8. 8. AI increases the accessibility of healthcare for everyone.

### Domain 3: Concerns About AI

1. 1. I am worried about the privacy of my personal information when using AI tools.
2. 2. I feel that AI may give incorrect advice in critical situations.
3. 3. I fear becoming too dependent on AI technologies.
4. 4. AI cannot understand human emotions like a nurse or doctor can.
5. 5. I am concerned about technical failures in AI systems.
6. 6. Using AI may reduce human interaction in healthcare.
7. 7. AI could replace human jobs in healthcare.
8. 8. I feel uncomfortable relying solely on AI for health decisions.

Scoring Interpretation:
• Positive Attitude: 81–120 points
• Neutral Attitude: 41–80 points
• Negative Attitude: 0–40 points
